# Extremely Elongated Cervix in an Adolescent Girl: Literature Review and Report of a Rare Case

**DOI:** 10.7759/cureus.24168

**Published:** 2022-04-15

**Authors:** Vibha Rani, Dharmendra K Pipal

**Affiliations:** 1 Gynaecology and Obstetrics, All India Institute of Medical Sciences Gorakhpur, Gorakhpur, IND; 2 General, Colorectal, and Minimal Access Surgery, All India Institute of Medical Sciences Gorakhpur, Gorakhpur, IND

**Keywords:** fertility, uterine descent, pelvic organ prolapse (pop), nulliparous, congenital elongation of cervix, adolescent

## Abstract

Pelvic Organ Prolapse (POP) is defined as the descent of one or more of the pelvic organs from their normal position. This is commonly associated with multiparity, postmenopausal status, and obesity. Most of the cases present as uterine descent with or without cystocele, rectocele, or enterocele. But, the descent of pelvic organs in adolescent and young, nulliparous women is an uncommon presentation.

We report a case of a 19-year-old girl with extreme elongation of the cervix without uterine descent. Uterus size was normal, no adnexal abnormality was there. The patient was apprehensive about her future fertility and pregnancy outcome.

This is a rare case as it has not been reported in the preceding three decades of literature searches and poses a challenge in management decisions because we must consider future fertility while restoring normal anatomy.

## Introduction

Pelvic Organ Prolapse (POP), which is common in the elderly, has a 40% prevalence in women over the age of 45, with 11-20% requiring surgical intervention, and has a poor quality of life [1]. The normal length of an adult non-pregnant cervix is about 2.5 cm to 3.0 cm. Isolated cervical descent with a normally positioned uterus is found in the case of true cervical elongation, which is a form of congenital elongation of the cervix and is a rare presentation in adolescence and young women.

The incidence of juvenile nulliparous uterine prolapse due to inherent congenital weakness of pelvic support is reported to be 1.5-2% [2,3]. The incidence of nulliparous prolapse in India is about 1.5-2% and 5-8% in young parous women [4]. In the pediatric and adolescent age group, this condition is found to be associated with congenital spinal defects, such as meningocele and myelomeningocele, bladder exstrophy, malnutrition, chronic lung disease, and heavy manual labour [5].

Cervical elongation is defined as the presence of cervical length >3.38 cm or cervix to corpus ratio > 0.79 [6]. Ibeanu and colleagues clinically defined cervical elongation as a C-to-D distance ≥ 8 cm* *[7]. Two possible mechanisms for this condition could be either inherently longer cervix or downward traction due to developing uterine prolapse causing cervical elongation [8,9].

Uterus-sparing surgery such as the Manchester-Fothergill procedure (MFP) and the Sling procedure using a minimally invasive approach are preferred for reproductive-age POP patients and offer the potential for preserving fertility.

## Case presentation

A 19-year-old adolescent girl presented in the Gynaecology OPD with a complaint of something coming per vaginum for the last six months, with the size of protruded mass increasing during defecation. There was no history of sexual contact, lifting heavy weights, chronic cough or constipation. She attained menarche at the age of 13 years and her menses were regular, cyclical with average flow and duration. There was no associated urinary complaint.

**Figure 1:**
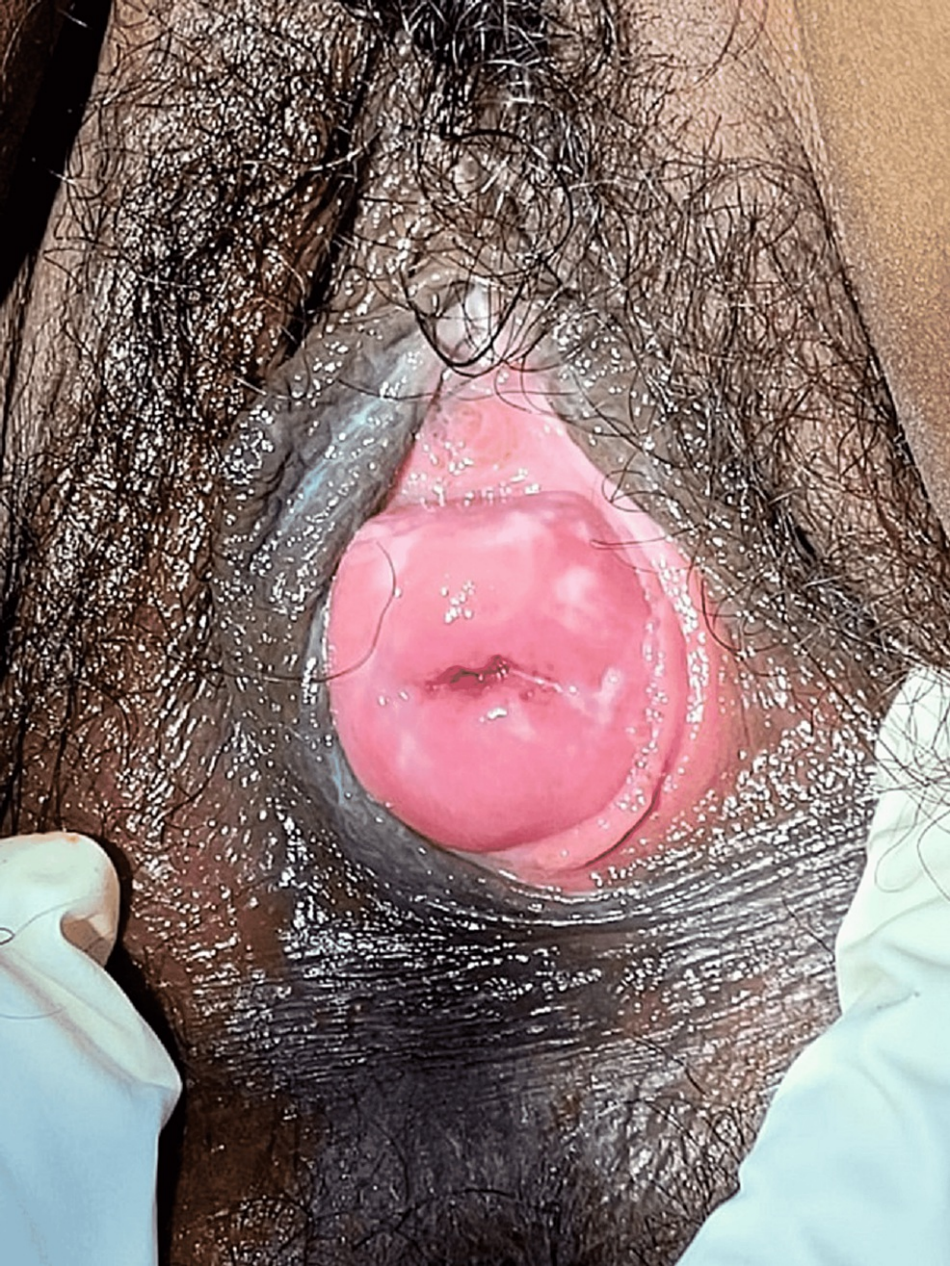
Extremely elongated cervix protruding through introitus.

On examination, third-degree cervical descent was noted (Figure-1), cervix was hypertrophied and elongated, firm in consistency and cervical length was approximately 6 cm, and all the fornices were felt. The uterus size was found to be normal.

**Figure 2:**
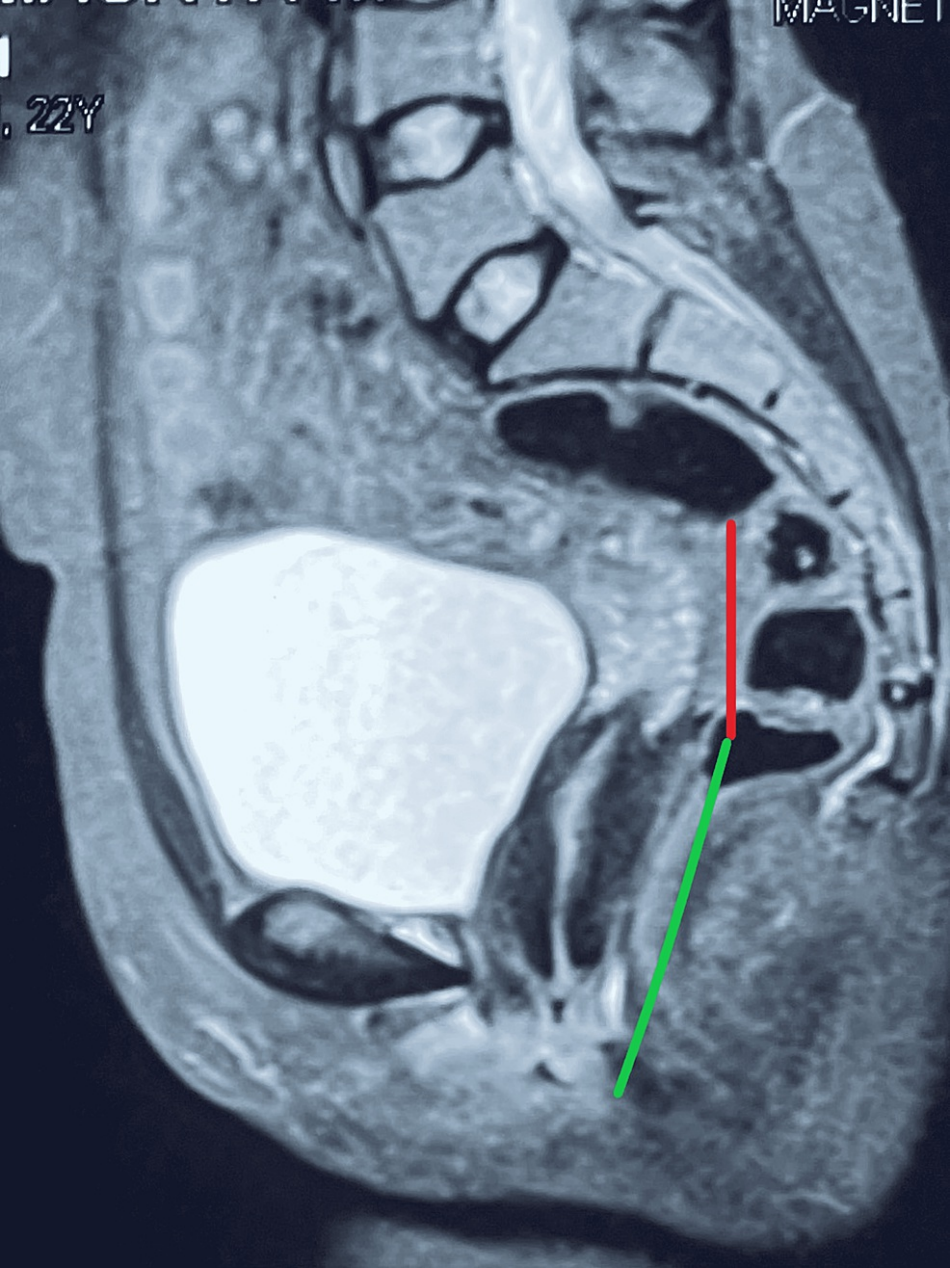
MRI pelvis showing a normal-sized uterus and an elongated cervix denoted by red and green lines, respectively.

MRI pelvis showed an elongated cervix with a length measuring 5.9 cm, bulging into the vagina inferiorly. The uterus was of normal size and in a normal position, and no abnormality in the adnexa was noted (Figure 2).

## Discussion

A cervical length of more than 33.8 mm is defined as cervical elongation and its pathophysiology is not yet clearly understood [5]. A review of 90 cases of elongation of the cervix presented 11 cases of congenital elongation of the cervix between the ages of 16-25 years, all patients had complained of something coming out per vaginum, and four had difficulty during coitus. All patients were treated by amputation of the cervix, four out of 11 treated patients conceived and had normal pregnancy outcomes [10]. 

Old age, multiparity, congenital weakness of pelvic floor muscles, prolonged labour, instrumental vaginal deliveries, birth trauma, chronic cough, genetic factor, smoking, prior surgery, collagen disorders including myopathies are a few well-known aetiological factors of POP [9].

Conservative management with ring pessary insertion may be considered if surgery is not possible or the pessary is ineffective in cases of uterocervical descent. There is no gold standard surgery to correct POP due to variable combinations of presentation. Different uterus preserving techniques using slings such as sacral cervicopexy or transvaginal sacrospinal fixation provide excellent repair and the possibility of childbearing [11]. 

A uterus-preserving technique, the Manchester-Fothergill procedure (MFP), is being performed for more than a century. There are several advantages of MFP including preservation of pelvic integrity, minimal blood loss so lesser hospital stay and morbidity, and also less recurrence rate [1]. Uterine preservation has a positive impact on women’s life including sexuality, body image, and self-esteem [1].

In sling procedures, Indian gynaecologists had contributed to developing conservative surgical treatment options with preservation of reproductive functions such as Shirodkar’s Sling and vaginal prolapse operation, Purandare’s Cervicopexy including its modified cervicopexy, Khanna’s Sling operation, Soonawala’s Sling operation and Nadkarni’s Sleeve Excision Anastomosis for cervical elongation [4]. Modified Gilliam-Doleris hysteropexy is a technique useful in juvenile nulliparous POP patients [3].

A systematic review described no difference in outcome among various sling procedures and vaginal hysterectomy [1]. With the exception of the MPF, uterine preservation is supported by lesser hospital stay [1,12]. The fertility rate following the MFP drops by 21-33% [13].

A case series studied causes of primary infertility in 3520 patients, 17 patients had extra-long vaginal cervix measuring 4.0 cm or more. Twelve out of 17 patients were treated by amputation of the cervix and 9 (75%) had successful pregnancy within 18 months while the rest five patients remained infertile and were not operated [14]. 

Manchester-Fothergill procedure is considered the fertility-sparing surgery in young women with extra-long cervix but the true incidence of successful pregnancy after the procedure is not known, probably due to the rarity of the condition. In our case, we had planned the Manchester-Fothergill procedure, but the patient is too young and apprehensive, so after having a detailed discussion, the patient and her family have decided to postpone any intervention till she marries.

## Conclusions

Congenital cervical elongation without uterine descent is an uncommon finding in an unmarried, nulliparous woman presenting as third-degree cervical descent, which creates apprehension for the patient and presents challenges for the Gynaecologist in deciding on the suitable uterine preserving surgeries. There should be a detailed discussion on future fertility issues and pregnancy outcomes before opting for the operative procedure in such patients. 
